# Detection of Gait Abnormalities for Fall Risk Assessment Using Wrist-Worn Inertial Sensors and Deep Learning

**DOI:** 10.3390/s20185373

**Published:** 2020-09-19

**Authors:** Ivana Kiprijanovska, Hristijan Gjoreski, Matjaž Gams

**Affiliations:** 1 Department of Intelligent Systems, Jožef Stefan Institute, 1000 Ljubljana, Slovenia; matjaz.gams@ijs.si; 2 Jožef Stefan International Postgraduate School, 1000 Ljubljana, Slovenia; 3 Faculty of Electrical Engineering and Information Technologies, Ss. Cyril and Methodius University, 1000 Skopje, North Macedonia; hristijang@feit.ukim.edu.mk

**Keywords:** fall risk assessment, balance deficit, gait abnormalities, information fusion, smartwatch, inertial sensors, deep learning

## Abstract

Falls are a significant threat to the health and independence of elderly people and represent an enormous burden on the healthcare system. Successfully predicting falls could be of great help, yet this requires a timely and accurate fall risk assessment. Gait abnormalities are one of the best predictive signs of underlying locomotion conditions and precursors of falls. The advent of wearable sensors and wrist-worn devices provides new opportunities for continuous and unobtrusive monitoring of gait during daily activities, including the identification of unexpected changes in gait. To this end, we present in this paper a novel method for determining gait abnormalities based on a wrist-worn device and a deep neural network. It integrates convolutional and bidirectional long short-term memory layers for successful learning of spatiotemporal features from multiple sensor signals. The proposed method was evaluated using data from 18 subjects, who recorded their normal gait and simulated abnormal gait while wearing impairment glasses. The data consist of inertial measurement unit (IMU) sensor signals obtained from smartwatches that the subjects wore on both wrists. Numerous experiments showed that the proposed method provides better results than the compared methods, achieving 88.9% accuracy, 90.6% sensitivity, and 86.2% specificity in the detection of abnormal walking patterns using data from an accelerometer, gyroscope, and rotation vector sensor. These results indicate that reliable fall risk assessment is possible based on the detection of walking abnormalities with the use of wearable sensors on a wrist.

## 1. Introduction

Falls are one of the most prevalent issues that affect the lives of elderly people and represent a significant public health problem [1]. In people older than 65 years, they lead to not only physical injuries but also psychological consequences that reduce their independence and decrease the quality of their life [2]. Falling incidents also result in severe public health care expenses, including the cost of hospitalization and rehabilitation [3], and can yield fatal outcomes as well. Due to the ongoing demographic change and aging of the population, fall-related problems are expected to further increase in the near future. Therefore, there is a great need for accurate screening tools for timely identification of those at risk of falling, to target appropriate fall prevention strategies.

Clinical research has shown that falls are a consequence of complex interactions between multiple intrinsic factors, such as cognitive difficulties, sensory deficits, and mobility problems [4]. Among them, the most commonly reported risk factors that lead to falls are balance deficit and gait impairment [5,6,7]. Most changes in gait for elderly people are related to underlying medical conditions. In fact, gait abnormalities are one of the first signs of an underlying disease. Therefore, early detection of an abnormal gait and balance disorder may predict future falls, and appropriate intervention may prevent or at least ease them. 

Existing clinical fall risk assessment sessions that include gait analysis, are performed by professionals in specialized laboratories that utilize costly equipment. This significantly limits the testing location and frequency, and the derived information may not precisely reflect the gait in real- life conditions due to the limited time of tests and the rush of adrenalin. Recent advancement in personal wearable devices makes them an attractive alternative approach for fall risk assessment, which can reduce the cost and considerably simplify the fall risk assessment procedure. At present, such wearable devices combine computational abilities and a variety of sensors that offer the benefit of continuous and real-time monitoring of gait. Moreover, they can be commonly carried by the user most of the day with minimal discomfort, thus providing regular insight into the risk of falling during daily living. Smartwatches are increasingly popular, mainly because people are accustomed to wearing watches, which makes the wrist one of the least intrusive locations to wear a device. However, developing a method for a wrist-worn device that makes use of sensor data for the successful detection of gait abnormalities is quite challenging and has not yet been validated in terms of fall risk assessment.

In this study, we show that non-invasive smartwatches or similar wrist-worn devices can be used in combination with deep learning (DL) methods to detect balance deficits and human gait alterations that are related to fall risk. In particular, we propose here a deep neural network (DNN) which integrates convolutional and bidirectional long short-term memory (LSTM) layers for successful learning of spatiotemporal features from multiple sensor signals. To aggregate the complementary groups of learned features from different sensor signals, the proposed method utilizes a feature-level fusion with the aim of improving gait abnormalities detection.

The highlights and contributions of our work can be summarized as follows:

1. A thorough review of the existing approaches for fall risk assessment that utilize wearable sensors and machine learning (ML) methods is presented here, also highlighting their current limitations.
2. A preparation of a dataset with realistic parameters of normal and abnormal walking patterns for 18 subjects. The inertial gait data were acquired using non-invasive smartwatches, worn on both wrists. This body location allows unobtrusive, continuous gait monitoring during daily routines and has not yet been explored in terms of fall risk assessment.
3. A novel DL method for gait abnormalities detection for wrist-worn devices that can be used at a user’s convenience during everyday life activities is proposed. It serves as proof of concept that wearable sensors can be used for reliable detection of balance deficit and gait abnormalities without the need for the patient to be in a clinical setting. To the best of our knowledge, this is the first study that employs a DL-based method for wrist-worn devices that detects gait abnormalities related to fall risk.
4. An analysis of the applicability of convolutional and bidirectional LSTM layers in a multi- channel DNN for learning adequate features from raw sensors signals is carried out here, removing the need of manual feature extraction and incorporation of particular domain knowledge.
5. An extensive evaluation of the proposed DL method is carried out here, including: (i) A comparison of the method’s performance using data from a single sensor and data from multiple sensors; (ii) a comparison of multi-sensor information fusion at various levels (data- level, feature-level, decision-level); (iii) a comparison of the proposed method with two classical ML methods, as well as a convolutional neural network (CNN) and LSTM network; (iv) a comparison of the performance of the method on the dominant and non- dominant wrist; (v) an analysis of the effects of changing the decision probability threshold of the proposed DNN when interpreting the predictions on its performance in terms of sensitivity and specificity.
6. A discussion about the results, efficiency, and significance of the proposed method, and its potential use in a free-living environment.

The paper is organized as follows: In Section 2, we discuss the related approaches serving as alternatives to existing clinical fall risk assessment, focusing on studies that utilize wearable sensor data and ML methods. In Section 3, we present the details on the collected dataset, the equipment used in the collection procedure, and explain the data preprocessing steps. In Section 4, we describe the employed methodology and the proposed DNN. Section 5 describes the evaluation setup and the comparison methods used in the study. The evaluation results are presented and discussed in Section 6. The paper is concluded in Section 7 with final remarks.

## 2. Related Work 

Recent technological progress has led to the development of various devices that allow for continuous human gait monitoring in free-living environments. Current sensor-based gait analysis systems are designed using either external sensors, such as cameras [8] and pressure sensors [9,10], or wearable sensors. However, because of the ability of the wearable sensors to provide a reliable insight into an individual’s gait quality in the least obtrusive way, they are becoming the most attractive approach for gait analysis and fall risk assessment. 

In general, the steps in ML methods that utilize wearable sensor data are preprocessing of the acquired signals, feature extraction from segments of the signals, and training of a model fed with those features. Therefore, the existing studies in the field of fall risk assessment from wearable sensor data mainly focus on the engineering of optimal features. The extracted features are given as an input to different ML algorithms for the prediction of fall occurrence, estimation of fall risk, or identification of gait abnormalities. The most frequently used ML algorithms employed in this domain are decision trees (DT) [11,12,13,14], support vector machines (SVM) [15], and random forest (RF) algorithms [16,17]. 

Recent studies have investigated the predictive value of various parameters extracted from inertial sensor data. Some of the most commonly extracted features from the sensor signals are from the time and frequency domains. They include the variance, mean, energy, autocorrelation, and dominant frequencies of the inertial sensor signals. Furthermore, some biomechanical features, such as gait stability, variability, and smoothness [18,19], and also turn mean duration [20], have been revealed as effective predictors for future falls. Gait characteristics extracted from inertial sensor data, such as stride length, clearance, stance and swing time for each stride, cycle time, cadence, and similar additionally improve the accuracy of fall risk classification [21]. These comprehensive sets of features are mainly extracted from tri-axial accelerometers and gyroscopes located at various locations on the body, from which the most exploited are the shanks, spine, head, pelvis, and feet [22,23]. Nevertheless, the estimation of most of these features often requires additional event detection and the incorporation of professional expertise to efficiently exploit the wealth of information that has been collected. Moreover, the manual extraction of features for ML-based systems is typically prone to bias due to the complex structure of sensor data collected from inertial measurement units (IMUs). 

On the contrary, DL allows models consisting of a number of processing layers to learn high- level features and data representations directly from raw sensor data. This ability of the DL architectures removes the need for manual extraction of features, which requires the integration of appropriate domain knowledge and expertise [24]. Due to this advantage, DL methods are becoming increasingly popular in the research community. They have been utilized in numerous areas, where they have provided findings that are comparable, or in certain cases, superior to those of human experts. DL has already infiltrated many domains of health informatics [25]. Existing research results have demonstrated its high capabilities and effectiveness in gait and behavior recognition [10,26,27]. However, its efficiency in the field of fall risk assessment has not been thoroughly explored. Therefore, in this work we explore the effectiveness of DL methods in gait abnormalities detection for fall risk assessment. One of the first attempts for the employment of DL methods for fall risk assessment was presented in [28]. The authors utilized a LSTM network, CNN, and a combination of a CNN and LSTM network to model fall risk based on accelerometer data from a sensor worn on the lower back. Another study that utilized DL methods is [29], where the authors worked with IMU signals from sensors attached to the feet of the subjects. They explored bidirectional LSTM networks that incorporated sequences of spatiotemporal gait parameters, as well as raw inertial data, to classify high fall risk and low fall risk patients. Similarly, in [9], LSTM networks were employed to classify artificially induced gait alterations from sensors worn inside the shoes. All of these studies show the potential of DL methods for fall risk assessment using wearable sensor data, achieving an accuracy of 76–82%. However, none of these studies have explored the wrist as a potential body position for gait analysis and gait abnormality detection for fall risk assessment. Indeed, the sensor signals collected from the wrist are more prone to noise compared to other body locations, for example the waist or torso. The main reason for this is that the hand is generally the most active part of the body and makes more irregular movements. This makes the gait analysis with data from a wrist-worn device extremely challenging. However, gait monitoring using a wrist-worn device has an advantage over other body positions in terms of obtrusiveness. The wrist is considered as the least obtrusive position to wear a device for longer periods of time, with minimal discomfort and without affecting day-to-day activities of the user. It therefore allows for continuous monitoring of gait and provides regular insight into the user’s risk of falling during daily living.

Considering the lack of evidence supporting the feasibility of fall risk assessment with sensors worn on the wrist, which has been considered as a highly desirable body position because of its superior user compliance, in this paper we propose a DL method that utilizes smartwatch sensor data for easy-to-implement, objective, and accurate fall risk assessment.

## 3. Dataset

### 3.1. Dataset Collection

**Figure 1:**
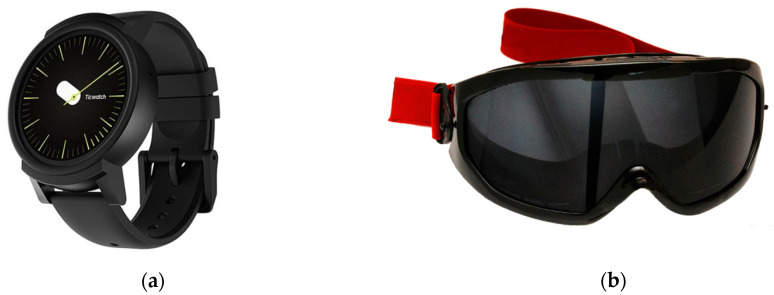
Equipment for data collection. (**a**) Mobvoi TicWatch E; (**b**) impairment glasses.

In the absence of an available dataset containing inertial sensor data collected with wrist-worn devices adequate for gait abnormalities analysis, we decided to conduct our own data collection procedure. For this purpose, 18 subjects were selected (8 males, 10 females, aged 19–54). Each subject wore two commercial smartwatches (one on each wrist), Mobvoi TicWatch E (Figure 1a), running on a Wear OS operating system, and impairment glasses (Figure 1b) [30].

First, we developed an application that collects a comprehensive set of data from the available sensors on the smartwatches, namely, the accelerometer, gyroscope, magnetometer, and rotation vector sensor. This was carried out at a sampling frequency of 100 Hz. The data collection sessions were synchronized between both devices and labeled using our designed application. Next, we developed a general procedure for the participants to follow during the data collection process. They were instructed to walk back and forth along a straight path of 15 m. Two scenarios were performed by all subjects included in the study, namely, a regular gait and a simulated abnormal gait. In the “regular gait” session, participants walked at a comfortable pace along the 15-m path, where they performed a so-called natural gait. In the simulated “abnormal gait” session, participants walked along the same path while wearing impairment glasses (Figure 1b). These glasses were used to simulate the effects of impairment, such as reduced alertness, a balance deficit, slowed reaction time, visual distortion, alteration of depth and distance perception, reduction of peripheral vision, and a lack of muscular coordination. All these things are related to disturbances in gait in elderly people, and their occurrence correlates with an increased risk of falls [31]. The participants were advised not to continue if they felt that they could not safely walk in a straight line unassisted (while wearing the impairment glasses), and a person was walking alongside (not touching) to prevent a potential fall if needed. Each participant repeated the 15-m walk a total of ten times, with five “regular gait” sessions and five simulated “abnormal gait” sessions. A total of 140 min of gait data were recorded.

### 3.2. Data Preprocessing

**Figure 2:**
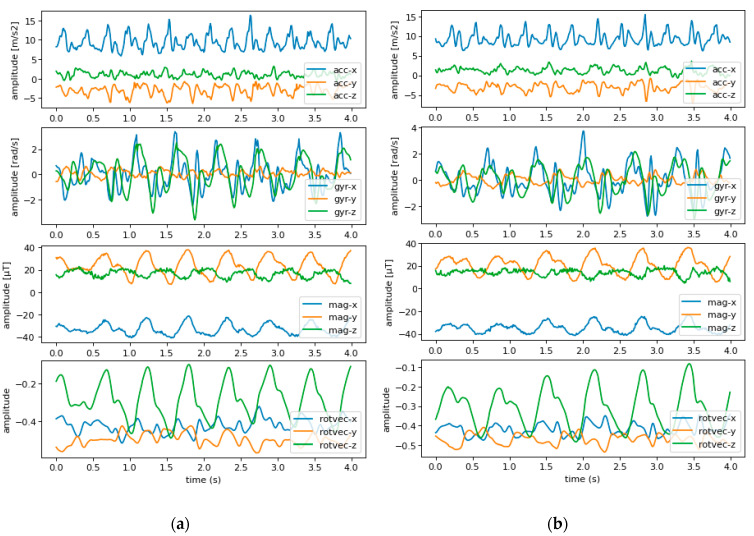
An example of two motion samples of 8 s from the smartwatch worn on the right wrist of one subject. (**a**) Normal walk; (**b**) abnormal walk. Acc—accelerometer, gyr—gyroscope, mag—magnetometer, rotvec—rotation vector.

The data collected from both sensing nodes (two smartwatches worn of the left and right wrist) contained 12 signals in total, i.e., three signals from each of the following sensors: Accelerometer, gyroscope, magnetometer, and rotation vector sensor (Figure 2). The data were initially sampled using a 100 Hz sampling rate for all sensors. However, a high sampling frequency could significantly affect the computational time of the method, due to the large amount of data that need to be processed. Moreover, the practical implementation of the method on a smartwatch would lead to excessive power consumption. To mitigate these issues, the data were first downsampled to 50 Hz. This sampling rate could save power and reduce the amount of data while keeping relevant signal information that is of interest to the gait analysis task [32]. 

The collected IMU data were contaminated with motion artifacts, along with the gait information. Since gait sensor data for normal walking contain frequency components in the range of 0.5 to 3.5 Hz, a 10-th order Butterworth band-pass filter was applied to extract the required frequency components from the IMU signals [33]. 

Next, we utilized a data augmentation technique. Data augmentation leverages limited data by transforming existing samples and creating new ones while maintaining the correct label. It is a widely used method for reducing the overfitting of DNNs caused by limited training samples. Some of the most commonly used data augmentation methods for IMU data include time-warping, scaling, and rotation [34]. Among these, we only utilized rotations as a data augmentation technique, since time-warping and scaling are more prone to altering the correct label for the sensor signal segments. Given that we were working with a wrist position for the sensors, there are limited possibilities for significant rotations of the device in real-life situations. Therefore, we chose to limit the rotation to the most probable case, where we applied 180 degrees rotation around the vertical axis to simulate reverse sensor placement, for example, if the smartwatch (or another wrist-worn device) is placed the other way round on the wrist. Employing this technique resulted in a doubling of the data samples for both sensor locations (left and right wrist).

The next step in the data preprocessing phase included scaling and standardization of the data. Many ML algorithms, especially DL algorithms, perform better and converge faster when the input signals are on a relatively similar scale or close to normally distributed. Therefore, we rescaled the data in such a way that the mean of the values was 0 and the standard deviation was 1. Such scaling was done signal-wise, i.e., independently for each sensor axis.

The last preprocessing step was the selection of an appropriate window size, which is used to split the signals from the sensors into segments. Employing this technique allows for perturbation of the temporal position of gait events within a window. Hence, it prevents an ML method from learning a class-specific characteristic in a segment based on its arbitrary temporal location. Longer windows usually contain more data regarding the walking patterns and are expected to enable higher classification accuracy. On the other hand, shorter windows allow for reduced resources and energy needs [35], which is preferable for methods intended for wearable devices. The optimal window size in our experiments was determined empirically. Eventually, we chose a window size of 8 s, with a 2- second overlap between consecutive windows for further experiments.

## 4. Methodology

DL is part of ML and is based on artificial neural networks [36]. DL allows deep architectures consisting of many processing layers to model complex non-linear relationships and learn data representations with multiple levels of abstraction. DNNs have been applied in a wide range of fields and have demonstrated an excellent capability of representation learning over many different applications. The most popular variations of DNNs which have been most widely used are CNNs and LSTM networks.

### 4.1. Convolutional Neural Network (CNN)

CNNs are DL architectures that can learn a hierarchy of features through convolutional and activation layers [37]. CNNs perform a convolutional operation between the input data and various filters in the convolutional layer, and an activation unit is utilized to generate the output features. Convolutional layers are comprised of a number of convolutional kernels (filters), each of which produces one feature representation of the corresponding input. The convolutional operation can be represented by Equation (1):

(1) $$O_{k}=f\left(W_{k}\times X\right)$$

where X is the input data, $W_{k}$ is the k-th convolutional kernel (filter), f is the nonlinear activation function, which performs the transformation of the output of the convolutional layer, and $O_{k}$ is the k-th feature matrix. The number of filters and their size are the crucial hyper-parameters that should be determined for the convolutional layers. When one-dimensional convolutional layers are considered [38], the convolution operation between the filter and the input data outputs a single scalar value. Moving the filter along the input sequence generates the feature vector after each convolutional layer. In general, CNNs are capable of learning characteristic patterns of the raw data and can be very effective at capturing spatial features from sensor signals. 

### 4.2. Long Short-Term Memory Networks (LSTM)

**Figure 3:**
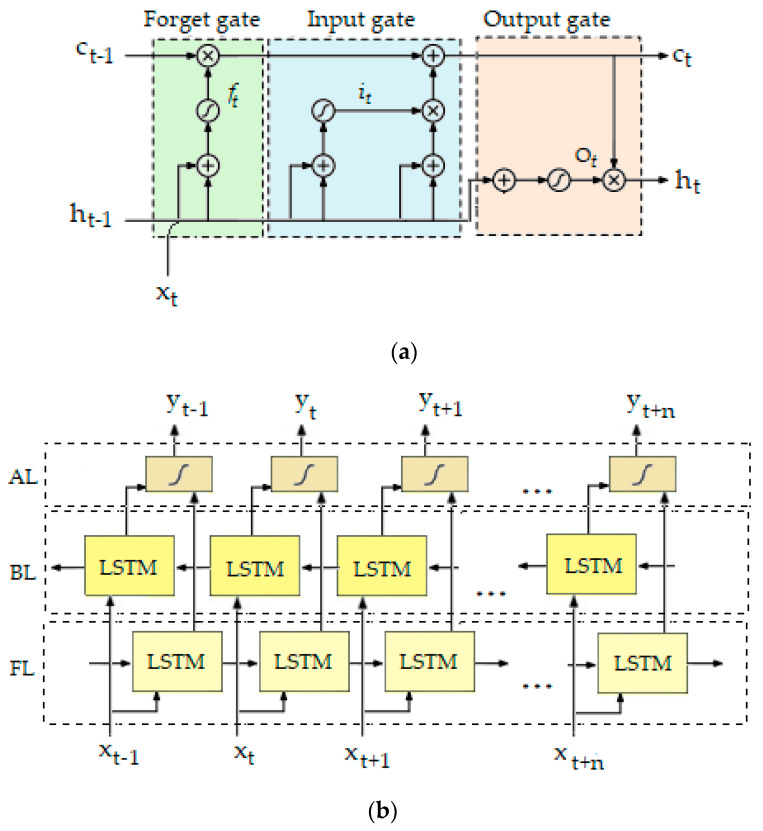
(**a**) Diagram of long short-term memory (LSTM) cell consisting of forget gate (f_t_), input gate (i_t_), and output gate (o_t_) which control the activation of the cell (c_t_) and the output of the cell (h_t_); (**b**) bidirectional LSTM. FL—forward layer, BL—backward layer, AL—activation layer.

LSTM networks are DL architectures based upon recurrent neural networks (RNNs), specifically developed to improve the modeling of complex time-series data without being affected by the main problem attributed to classical RNNs, namely, gradient vanishing. The LSTM structural cells are comprised of three “gates”, namely, an input gate, a forget gate, and an output gate, which control the status of the memory cell (Figure 3a). They have the ability to remove or add information to the cell state, thus allowing optimal passage of information through the network. LSTM networks process signals in a recursive manner and therefore have the potential to capture their sequential and temporal dependencies [39].

Moreover, bidirectional LSTM networks, as an extension of the typical unidirectional LSTM networks, can further enhance the performance of sequence classification models by connecting two hidden layers of opposite directions to the same output (Figure 3b). As opposed to classical unidirectional LSTM networks that are only able to access the previous dependency information of each specific timestamp, by adopting bidirectional LSTM network architecture, the past and future context can both be exploited to capture temporal information. The supplementary context added to the network results in more comprehensive learning of a particular problem. 

### 4.3. Proposed DNN Method

In this section, we describe our proposed method for multi-sensor time-series classification for the aim of gait abnormalities detection. The proposed end-to-end DL method integrates a CNN and bidirectional LSTM network. These two types of DNNs are able to extract essential features from the internal structure of sensor data in an end-to-end learning process, without the inclusion of particular domain knowledge. The main idea of using both CNNs and LSTM networks is to make full use of their complementary abilities for feature learning. Moreover, to aggregate the complementary groups of learned features, the proposed method utilizes feature-level fusion of multiple sensor signals. 

The DL architecture takes 8-s segments from various sensor signals as input, where each segment is associated with a corresponding normal/abnormal gait label. The segments from each sensor signal are then parallelly processed by a CNN and a bidirectional LSTM network. More precisely, the DL architecture consists of a dedicated channel for each sensor signal, consisting of a CNN and a bidirectional LSTM network.

The CNN consists of two one-dimensional convolutional layers, each of them followed by a batch normalization (BN) layer [40] (for reducing the internal covariate shift) and a rectified linear unit (ReLU) activation layer [41]. These two convolutional blocks (convolutional layer + BN + ReLU) are followed by a max pooling layer [42]. It reduces the revolution of the learned features, consolidating them to only essential elements, thus improving the robustness of the extracted patterns. Both convolutional blocks learn 10 filters with a kernel size of 5. The bidirectional LSTM network consists of two bidirectional LSTM layers, containing 20 and 10 nodes, respectively, followed by a dropout layer [43]. 

**Figure 4:**
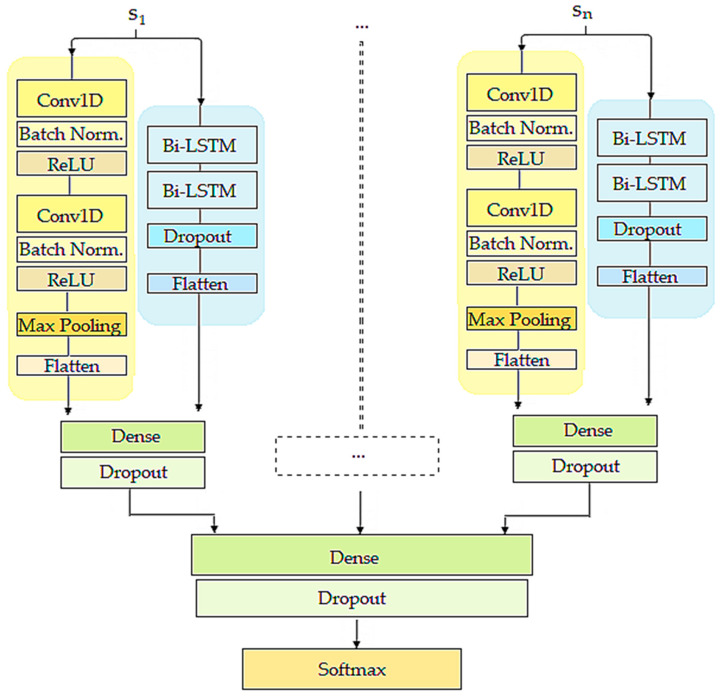
Proposed architecture for gait abnormalities detection—each sensor signal is processed by a dedicated channel consisting of a convolutional neural network (CNN) and a bidirectional long short-term memory (LSTM) network. The architecture utilizes feature-level fusion of multiple sensor signals.

Both groups of learned features (spatial and temporal) for each signal are further fused into a fully-connected (dense) layer. This feature-level fusion projects the complementary temporal and spatial feature representations for each sensor signal into the same common space, thus enabling the further layers to discover internal characteristics of the signal structure from two different aspects. The outputs of these fully-connected layers are once more fused into another fully-connected layer. This layer is shared among all sensor signals, which means that it interprets the independent sets of weights respective to each modality. In other words, this layer takes into account all features that are learned for each sensor signal incorporated in the network architecture. To avoid overfitting, a dropout layer is also used after each fully-connected layer. Eventually, the final output of the network is provided by a softmax layer [44], which returns a class probability for each of the two classes. The overview of the proposed DL method is displayed in Figure 4.

The model was trained by minimizing the binary cross-entropy loss function. The data were re-shuffled at every epoch to avoid bias errors due to the training data order. An Adam optimizer [45] was used to optimize the weight of the network. The learning rate of the optimizer was set to be 0.001, and the batch size was set to 256. The method was trained with the early stopping technique [46] and the maximum number of training epochs was set to 30. The number of maximum training epochs was chosen experimentally, as it showed that for a larger number of training epochs the generalization error increased. The proposed DL method was implemented using TensorFlow [47].

The learning phase of one epoch in the DNN takes about 8 min on a single NVIDIA GeForce RTX 2060 GPU (by Gygabyte Technology), while the classification on the test data takes about 13 s for 200 instances on average.

## 5. Experimental Setup

### 5.1. Comparison Methods

To confirm the performance of the proposed method and further test the effectiveness of particular modules implemented in it, we employed several methods for comparison.

**Figure 5:**
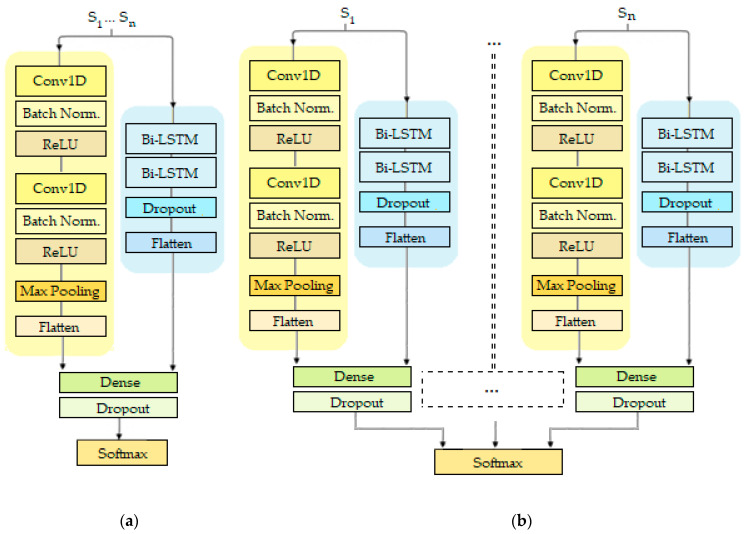
Deep neural networks (DNNs) which implement different information fusion methods. (**a**) Data-level; (**b**) decision-level.

The feature-level fusion implemented in the proposed method was compared against two other information fusion methods, namely, data and decision-level fusion [48]. The idea behind data-level fusion is to incorporate all possible information that various sensors generate as a single time-series input for the DNN. In other words, instead of processing each sensor signal in a dedicated channel (as the proposed DNN does), with data-level fusion, the DNN is fed with a single time-series, where each sensor signal is treated as an additional dimension of the time-series. The structure of the DNN that implements data-level fusion is shown in Figure 5a. The DNN that implements decision-level fusion is structured similarly to our proposed DNN. However, instead of combining the separate sets of learned features respective to each sensor signal in a shared fully-connected layer, they are only combined as inputs for the final output layer [49]. The structure of the DNN that implements decision-level fusion is presented in Figure 5b. 

To further explore the usefulness of the fusion of two complementary groups of learned features, i.e., spatial and temporal features, a CNN and bidirectional LSTM network were independently applied to learn features from the raw data of IMU sensor signals. Consequently, only one group of features was learned for each sensor signal (spatial or temporal, respectively). Both of these DNNs have the same structure as the proposed DNN (in terms of processing layers and hyper-parameters), without the fully-connected layer that fuses the two complementary groups of features for each sensor signal.

Lastly, to prove the effectiveness of the proposed method in learning features from raw sensor signals, we compared it with classical ML methods that work with manually extracted features. We used two classical ML algorithms that are commonly utilized in this field, namely, SVMs [50] and RFs [51]. SVM is a classifier algorithm which is characterized by the usage of statistical learning theory to provide a hyper-plane in the feature space that divides the instances according to the class label. It uses a kernel function to transform feature vectors into higher dimensional space and deals with non-linearly separable data. RF is an ensemble classifier algorithm that fits a number of decision trees on various sub-samples of the dataset and outputs the majority class label from the constructed trees. It utilizes two random steps in the process of creating trees, namely, a random sampling of the training data points and a random choosing of a splitting feature, which make it robust to noise and outliers. To train these models, we extracted time-domain and frequency-domain features for each sensor signal. This procedure resulted in 396 features in total. The time-domain features were computed with the TSFRESH Python package and included the mean, standard deviation, median, maximum, minimum, mean absolute change, variance, kurtosis, skewness, among others. The frequency-domain features were calculated using the power spectral density (PSD) of the signal, based on the fast Fourier transform (FFT). They included the three largest magnitudes of the FFT components, the entropy of the normalized FFT components, and their energy [52,53]. The RF model was trained with 100 estimators with a gini criterion to measure the quality of the splits of the tree. The SVM model was trained with a radial basis function kernel and a regularization parameter with value 1.

### 5.2. Validation and Evaluation Metrics

To estimate the generalization accuracy of the methods, a leave-one-subject-out (LOSO) cross-validation technique was utilized. With this technique, the data are divided into N-number of folds, where N is the number of subjects in the dataset. Each fold is comprised of data from a single subject. Further, in each iteration of the LOSO cross-validation, data from one subject are used for testing the method, and the training data are comprised of the remaining N-1 subjects. This procedure is repeated until data from all subjects have been used as testing data. This validation technique rules out the possibility of the model to learn the subject’s identity by making sure that one subject’s data are not mixed into both the training and test sets. As a primary evaluation metric, we report accuracy, which denotes the ratio of the number of correct predictions over the total number of predictions. This metric is suitable because we have a fairly balanced dataset. For additional performance insight, sensitivity (true positive rate) and specificity (true negative rate) are also reported. Accuracy, sensitivity, and specificity are calculated as shown in Equations (2)–(4):

(2) $$Accuracy=\;\frac{TP+TN}{TP+FP+TN+FN}$$

(3) $$Sensitivity=\frac{TP}{TP+FN}$$

(4) $$Specificity=\;\frac{TN}{TN+FP}$$

where TP denotes true positives, TN denotes true negatives, FP denotes false positives, and FN denotes false negatives.

## 6. Experimental Results

In this section, we present the results from the experiments that were carried out. Section 6.1 presents the results obtained with the proposed method (DNN that integrates feature-level multi- signal fusion), as well as with DNNs that implement data-level and decision-level information fusion, achieved with the utilization of single or multiple sensor data. In Section 6.2, the performance of the proposed method is compared against two other DL methods and two classical ML methods, with various combinations of sensors data. Section 6.3 presents a comparison of the performance of the proposed method on the dominant and non-dominant wrist. Lastly, in Section 6.4, we analyze the effects of changing the decision probability threshold of the DNN when interpreting the predictions on its performance in terms of sensitivity and specificity. All results presented in this section are obtained using LOSO cross-validation.

### 6.1. Single vs. Multiple Sensors for Different Information Fusion Levels 

Table 1 displays the results achieved by using signals from a single sensor as input in a DL architecture, with varying levels of information fusion. 

If we compare the results for different information fusion levels, it can be noted that the chosen feature-level fusion DL architecture achieves the highest accuracy among the three architectures for each sensor. The highest accuracy is achieved with the gyroscope sensor data, specifically, 85.8% (89.9% sensitivity and 79.7% specificity), and the architecture achieves similarly high accuracy with the accelerometer data as well (83.7%). However, when the DL network is only fed with data from a magnetometer or rotation vector sensor, the results considerably decrease, especially in terms of the specificity. Regarding the information fusion level, decision-level fusion reports the lowest results in terms of overall accuracy, and also reports significantly lower specificity results for each sensor. 

We further explored the performance of the three DL methods (data-, feature- and decision-level information fusion) with a combination of data from multiple sensors. Given the four available sensors from the dataset, we made eleven sensor combinations, namely, accelerometer + gyroscope (AG), accelerometer + magnetometer (AM), accelerometer + rotation vector (AR), gyroscope + magnetometer (GM), gyroscope + rotation vector (GR), magnetometer + rotation vector (MR), accelerometer + gyroscope + magnetometer (AGM), accelerometer + gyroscope + rotation vector (AGR), accelerometer + magnetometer + rotation vector (AMR), gyroscope + magnetometer + rotation vector (GMR), and accelerometer + gyroscope + magnetometer + rotation vector (AGMR). The results from these experiments are shown in Table 2. The incorporation of data from multiple sensors into the DNNs results in higher accuracy when compared to the results achieved with the incorporation of single sensor data. For example, from the single sensor models, the highest achieved accuracy was 85.8% (with gyroscope sensor data). However, when the gyroscope data were combined with the accelerometer data, the same DL architecture (with feature-level information fusion) achieved 87.8% accuracy. Even more, using data from three sensors leads to even higher classification performance than using data from two sensors. In fact, the highest results were achieved with the feature-level fusion method (proposed method) for the sensor combination, using the accelerometer + gyroscope + rotation vector sensor (AGR) configuration and conferring 88.9% accuracy in the classification of normal and abnormal walking patterns. This combination also achieves high sensitivity, specifically, 90.6%, which is extremely important in identifying abnormal walking patterns related to high fall risk. Moreover, this sensor combination leads to the highest specificity score. The combination that utilizes all four available sensors (AGMR) achieves slightly worse results in terms of the overall accuracy (86.2%).

The results show that the chosen feature-level fusion of multiple sensors signals is more effective than fusion at an earlier or later stage and enhances the performance of gait abnormalities identification as compared to the other two levels of fusion (data-level and decision-level).

### 6.2. Evaluation of Comparison Methods

To further explore the usefulness of the proposed method, we compared it to two other DL methods, a CNN and bidirectional LSTM, and two classical ML methods, SVM and RF, which work with manually extracted features (see Section 5.1). For this comparison, we used data from the two sensors with which the single-sensor methods achieved the highest accuracy (see Table 1), and the two best two- and three-sensor combinations (see Table 2). The results from these experiments are presented in Table 3.

The proposed method, based on automatic feature learning and optimal fusion of two complementary groups of features learned with CNN and bidirectional LSTM network, achieves the highest accuracy among all other comparison methods for each sensor combination.

The comparison of the results achieved with the proposed network and the classical ML methods indicates that more valuable information about the gait balance and stability can be extracted with automatic feature learning from the raw sensor signals than with the manual extraction of time and frequency-domain features. With manually crafted features, both classical ML methods provide similar results. Moreover, classical ML algorithms achieve the highest accuracy with the same sensor combination as the proposed method, which suggests that these sensors provide the most valuable information for the detection of walking abnormalities, regardless of the way of feature extraction (automatic or manual).

The comparison of the results achieved with the proposed method and the other two DL methods shows that the combination of the CNN and LSTM network has a much better feature learning ability than a CNN or LSTM network when they are used independently. The presented results further prove the importance of the proposed feature-level fusion and incorporation of the two complementary groups of features (spatial and temporal). They allow the model to gain a more in-depth insight into the sensor signal structure, thus enabling it to distinguish normal and abnormal walking patterns better. Furthermore, the bidirectional LSTM network achieves higher accuracy when compared with the CNN, which implies that walking abnormality detection is more sensitive to time-related features and temporal dependencies give more valuable information about gait balance and stability. However, their combination with spatial features improves the overall accuracy of the method.

Additionally, to check whether the misclassification rate difference between our method and the other classifiers was statistically significant, we also utilized the McNemar’s statistical hypothesis test [54]. It is a non-parametric statistical significance test for paired observations that reports on the different correct or incorrect predictions between two methods that are being compared [55]. We compared our method’s performance to all other methods for the best sensor combination (AGR). The calculated p-value was lower than 0.05 for each comparison. This result shows that the methods not only make different errors but also have a different relative proportion of errors. In other words, we may state that there is a statistically significant difference in the predictions of the methods.

The presented results show the ability of the proposed DL method to automatically learn high- level features that are relevant for appropriate gait analysis directly from raw sensor data. It achieves higher accuracy in detection of gait abnormalities than all compared methods, without the need for complex manual feature engineering and professional expertise. Even more importantly, the results prove the relevance of the proposed method in the sense that DL methods based on IMU sensor data from a wrist-worn device are a promising alternative to current approaches and have a potential for real-life application. 

### 6.3. Method’s Performance on the Left (Non-Dominant) vs. Right (Dominant) Wrist 

All experiments so far utilized data from both the right and left wrist for training and testing. In this section, we analyze the performance of the proposed method on the left and right wrist separately to further test its performance in real-life scenarios. We want to see if the method performs equally well on both wrists and if training a method with data from only one particular wrist could improve the results. Moreover, real-life situations pose additional challenges that should be taken into account when considering the practical implementation of the method. One possible challenge that may occur is a method to be developed for the left (right) wrist and the user to wear the device on the right (left) wrist, or the person to be indiscriminately left- or right-handed [56]. Therefore, we took several combinations into account.

Table 4 shows the accuracy of the method for six train-test combinations: (i) Training on the right wrist and testing on the right wrist (right-right); (ii) training on the left wrist and testing on the left wrist (left-left); (iii) training on the right wrist and testing on the left wrist (right-left); (iv) training on the left wrist and testing on the right wrist (left-right); (v); training on both the right and the left wrist and testing on the right wrist ((right + left)-right); (vi) training on both the right and the left wrist and testing on the left wrist ((right + left)-left). 

The results show that the left-right and the right-left combinations achieve 79.9% and 82.0% accuracy, respectively, which is lower compared to the right-right (83.1%) and left-left combinations (85.3%). The presented results suggest that training a classification model for a particular wrist would not perform equally well if the wrist-worn device is worn on the other wrist. Even though the models trained with data from only one wrist achieve extremely high sensitivity levels, it is obvious that they tend to over fit to the “abnormal walk” class, hence the low specificity levels. If data from both wrists are included in the training set, this mitigates the problem of switching wrists. The inclusion of data from both wrists improves the accuracy, as well as the specificity of the right-right and left-left models, which implies that the initial training set consisting of data from both left and right wrist is the one that should be deployed in the final model. It is important to mention that the better performance of these models does not necessarily come because of the inclusion of data from both left and right wrist, but it is possible that it is only due to the inclusion of larger amount of training data. The performance of deep learning architectures generally gets better when more data is included in the training phase. However, to get an accurate picture of the real cause, further research needs to be done that will include additional data. 

Lastly, if we compare the last two columns of Table 4, it can be seen that the model trained with data from both the right and left wrist performs better for the left wrist, achieving 89.2% accuracy, as opposed to the right wrist with 88.7%. Also, the specificity is considerably higher for the left wrist (more than 5 percentage points). The classification model achieves a particularly high sensitivity score for the left wrist as well, which is of particular interest for the gait abnormality detection task. Since all subjects included in the dataset were right-handed, this suggests that the non-dominant hand brings more information regarding one’s walking patterns. 

### 6.4. Method’s Performance with Different Values of the Probability Threshold 

As mentioned above, the final output of the proposed DNN was provided by a softmax layer, which outputs a vector that represents the probability distributions of the potential outcomes. In other words, it returns the class probability for each of the two classes, i.e., normal or abnormal walking classes in our case. The class probability provides a measure of the certainty of a prediction. The decision to convert the probability into a class label was determined by a probability decision threshold. Namely, all output probabilities equal to or higher than the specified threshold were mapped into one class, and the probabilities lower than the threshold were mapped into another class. The default decision threshold for binary classification problems was 0.5.

We experimented with different values of this probability decision threshold when classifying a segment as normal or abnormal walking. With this experiment, we want to see if it is possible to reach higher sensitivity levels without significantly compromising the model’s performance in terms of the specificity or overall accuracy. Failing to detect abnormal walking patterns could have serious health ramifications, whereas misclassifying normal walking patterns as abnormal ones would only result in unnecessary precautions and interventions. Therefore, for our task it is extremely important to achieve high sensitivity levels in abnormal gait detection.

For this purpose, we experimented with changing the probability threshold parameter when classifying normal walking patterns. Namely, rather than using the standard probability threshold of 0.5, we examined the performance of the proposed DNN with the threshold set to 0.6, 0.7, 0.8, or 0.9 when classifying normal walking patterns. This means that the DNN would classify a particular segment as normal walking only if its class probability is higher than 0.6, 0.7, 0.8, or 0.9. Otherwise, the segment would be treated as an abnormal walk. In other words, if there is even a slight chance that the walk could be abnormal (0.4, 0.3, 0.2, or 0.1 class probability, respectively), the DNN would classify it as an abnormal walking segment. 

The results of this experiment can be seen in Table 5. We compare the results achieved on segment-level (as all experiments above) but also on trial-level. One trial is considered as one walking session performed by a subject along the 15-m-long path, back and forth (see Section 3.1). The label for each trial was calculated as the majority class of its corresponding segments. 

As expected, with the moving of the threshold, the sensitivity increased while the specificity decreased. Consequently, there was a slight change in the overall accuracy of the proposed method as well. However, the change in the classification accuracy on a segment-level was not considerable (<0.4 percentage points). On the other hand, if we observe the results achieved on the trial-level, an increase of 1 percentage point in the overall accuracy can be noted for the model with a probability threshold value of 0.8 (in comparison with the default model with a probability threshold value of 0.5). Moreover, the DNN with a probability threshold value of 0.8 achieves 3.3% higher sensitivity at the expense of the model’s specificity, which drops by 1.3%. When the probability threshold value was set to even higher value, such as 0.9, the overall model’s accuracy dropped and the specificity of the DNN considerably decreases by 5%. 

The conclusion from this analysis is that by changing the probability threshold it is possible to improve the method’s performance in terms of accuracy and sensitivity, without significantly compromising the method’s performance in terms of specificity. However, it should be noted that before making such adjustments of the probability decision threshold, one should precisely consider which misclassification cost is more critical for the given task.

## 7. Conclusions

In this study, we presented a novel DL method that detects human gait abnormalities with a wrist-worn device. The detected abnormalities were used here to assess the risk of falling. We prepared a dataset with realistic parameters of the normal and abnormal walking patterns of 18 subjects. The inertial gait data were acquired using non-invasive smartwatches, worn on both wrists. This body location offers the benefit of unobtrusive, continuous, and real-time gait monitoring during daily routines. 

The proposed IMU-based DL method integrates a CNN and bidirectional LSTM network for the successful learning of complex spatiotemporal features from multiple sensor signals. To aggregate the complementary groups of learned features from different sensor signals, the proposed method utilizes feature-level fusion. In the classification stage, the learned feature maps for each sensor signal were further combined as an input of a shared fully-connected layer that interprets the independent sets of weights respective to each modality.

The effectiveness of the proposed method was verified through the evaluation against comparison methods. The proposed feature-level fusion DNN achieved the highest accuracy in the detection of abnormal walking patterns, compared to the data-level and decision-level fusion DNNs, with data from single and multiple sensor signals. The highest results were achieved with a combination of accelerometer, gyroscope, and rotation vector data, conferring an accuracy of 88.9%, a sensitivity of 90.6%, and a specificity of 86.2%. These results are significantly better than the results achieved with single-sensor data and suggest that the incorporation of multiple sensor signals into the model leads to higher accuracy. Moreover, they show that the proposed feature-level fusion of multiple sensors signals is more effective than data-level and decision-level fusion and enhances the performance of gait abnormalities identification.

The comparison of the results achieved with the proposed method and the results achieved with a CNN and a bidirectional LSTM network further proved the effectiveness of the feature-level fusion of two complementary groups of features (spatial and temporal). The fusion of spatiotemporal features allows the model to gain a more in-depth insight into the sensor signal structures, which results in a better distinction of normal and abnormal walking patterns. Furthermore, the bidirectional LSTM network achieved higher accuracy than the CNN, which implies that walking abnormality detection is more sensitive to time-related features and that temporal dependencies give more valuable information about gait balance and stability.

The comparison of the results achieved with the proposed method and the results achieved with the classical ML algorithms showed the overall ability of the proposed DNN to learn effective feature representations from raw sensor signals. The automatically learned spatiotemporal features proved to be more informative about the gait balance and stability than the manually extracted time and frequency-domain features.

Additionally, we tested the performance of the method for the right (dominant) and left (non- dominant) wrist. The analysis showed that the left wrist, which was the non-dominant one for all subjects in our dataset, slightly outperformed the right wrist. Moreover, the results from the analysis showed that the best practical solution is to train a classification model with data from both wrists, regardless of which wrist the model is intended for. 

Lastly, we analyzed the effects of changing the decision probability threshold of the DNN when interpreting the method’s predictions on its performance in terms of sensitivity and specificity. We concluded that with changing the probability threshold, it is possible to improve the method’s performance in terms of accuracy and sensitivity without significantly compromising the method’s performance in terms of specificity. Achieving high sensitivity levels for abnormal walking pattern detection is extremely important for our task, since failing to detect abnormal walking patterns could have serious consequences. In contrast, misclassifying normal walking patterns as abnormal ones would only result in unnecessary precautions and interventions. 

Overall, the achieved results show that the proposed DNN is capable of the distinction of normal and abnormal walking patterns and is robust enough to cope with data from participants for which it had no prior knowledge. With an ever-aging population who requires increasing medical assistance, the results achieved in this study demonstrate the significant potential for diagnostics and elements of treatment to move from clinical settings to the home, reducing costs and the burden on both practitioners and patients. Furthermore, the results from this study indicate that detection of walking abnormalities and fall risk assessment can be done using wearable sensors and reveals the wrist as a potential fall risk assessment source. 

It is important to note that our method currently works on the assumption that the walking activity is already recognized and is used as input. We believe that this would not be a practical issue since numerous studies show that walking activity can be successfully recognized with a wrist-worn accelerometer [56,57]. Therefore, we envision our method working alongside an activity recognition method, which would first discriminate the period of walking, and afterwards would analyze the detected walking segments.

In our experiments, the gyroscope sensor was found to be the most effective sensor modality. In practice, this sensor is known to have a drift. Although the current measurements indicate that even without a special method to deal with the drift, the data enables detection with reasonable quality, this characteristic of the gyroscope sensor might be a potential limitation of the method, and its impact on the method’s performance will be further studied in the future.

Another limitation of the study is the fact that the current dataset only contains simulated abnormal gait. However, the presented results demonstrate the significant potential of the proposed approach and are a solid basis for further research on this topic. In fact, for future work, we plan to further evaluate the performance of the method in real-life conditions while users are performing their everyday activities. We wish to conduct an extensive study that will include data from aging adults as well, which will enable a systematic analysis of gait-related differences between diverse subject groups. Another idea that can be explored is the training of person-specific classification models. Moreover, we intend to attempt to further increase the classification performance of the method by combining it with a hidden Markov model, or similar, which would also take into account information on the temporal dependencies of sequential windows [58]. Lastly, we plan to incorporate energy-optimization techniques for conserving the battery power of the sensors, which is one of the main constraints on smartwatches and similar wrist-worn devices [59].

## Figures and Tables

**Table 1:** Average testing accuracy, sensitivity, and specificity achieved with a deep learning (DL) architecture with different information fusion levels (data-, decision-, and feature-level), and with single sensor data (accelerometer, gyroscope, magnetometer, and rotation vector sensor). Leave-one-subject-out (LOSO) evaluation (%).

Fusion Level		Sensor			
		Accelerometer	Gyroscope	Magnetometer	Rotation Vector
**Data**	**Accuracy**	83.1	85.6	67.5	70.5
	**Sensitivity**	85.5	89.8	93.2	86.9
	**Specificity**	79.4	79.1	28.1	45.5
**Decision**	**Accuracy**	78.3	81.2	63.2	61.7
	**Sensitivity**	89.8	87.7	82.2	73.7
	**Specificity**	60.5	71.2	36.3	44.8
**Feature** **(chosen)**	**Accuracy**	83.7	**85.8**	77.2	68.3
	**Sensitivity**	90.8	**89.9**	93.4	76.9
	**Specificity**	72.9	**79.7**	52.4	55.0

**Table 2:** Average testing accuracy (acc.), sensitivity (sens.), and specificity (spec.) achieved with a DL architecture with different information fusion levels (data-, decision-, and feature-level), and with multiple combinations of sensor data. LOSO evaluation (%). A—accelerometer, G—gyroscope, M— magnetometer, R—rotation vector sensor.

Fusion Level		Sensors										
		AG	AM	AR	GM	GR	MR	AGM	AGR	AMR	GMR	AGMR
**Data**	**Accuracy**	87.3	65.3	78.1	76.0	79.2	74.7	85.7	85.2	70.5	78.6	83.5
	**Sensitivity**	90.2	74.9	83.4	92.5	92.1	84.8	89.2	88.8	84.9	92.8	87.4
	**Specificity**	82.5	49.8	69.7	50.6	59.3	58.5	80.2	79.4	47.1	56.8	77.4
**Decision**	**Accuracy**	80.6	61.8	71.4	72.6	74.9	60.4	71.5	74.0	66.5	74.2	72.7
	**Sensitivity**	95.2	74.5	70.6	92.5	90.4	94.3	84.0	85.4	84.9	86.8	79.4
	**Specificity**	57.0	43.9	72.8	43.1	52.1	12.6	53.9	58.0	40.5	55.6	63.0
**Feature** **(chosen)**	**Accuracy**	87.8	79.6	79.3	83.7	85.1	63.9	88.3	**88.9**	78.1	82.7	86.2
	**Sensitivity**	91.9	88.8	82.0	88.6	89.6	95.7	81.7	**90.6**	89.1	89.9	87.6
	**Specificity**	81.2	64.7	74.8	76.2	78.2	12.5	82.8	**86.2**	60.4	71.7	84.1

**Table 3:** Average testing accuracy achieved with two classical machine learning (ML) methods, and three DL methods, for six sensors combinations. LOSO evaluation (%). A—accelerometer, G—gyroscope, M—magnetometer, R—rotation vector *.

Method	Sensor Combination					
	A	G	AG	GR	AGM	AGR
**RF**	82.8	81.6	83.5	82.4	83.4	84.1
**SVM**	81.3	81.6	81.8	81.2	82.2	84.0
**CNN**	77.5	79.1	80.9	77.5	76.8	85.2
**LSTM**	83.0	83.4	86.1	81.7	79.8	86.4
**Proposed**	83.7	85.8	87.8	85.1	88.3	**88.9**

* The results for the random forest (RF) and support vector machine (SVM) were achieved with manually extracted features, and CNN, LSTM, and the proposed method work with raw signals.

**Table 4:** Average testing accuracy (acc.), sensitivity (sens.), and specificity (spec.) for six train-test combinations. LOSO evaluation (%).

	Train-Test					
	Left-Right	Right-Left	Right-Right	Left-Left	(Right + Left)-Right	(Right + Left)-Left
**Accuracy**	79.9	82.0	83.1	85.3	**88.7**	**89.2**
**Sensitivity**	94.2	92.0	97.0	93.5	**91.8**	**89.4**
**Specificity**	56.7	65.9	60.5	72.2	**83.7**	**88.8**

**Table 5:** Average testing accuracy, sensitivity, and specificity for different values of the probability threshold on segment-level and trial-level. LOSO evaluation (%).

Threshold	Segment-Level			Trial-Level		
	Accuracy	Sensitivity	Specificity	Accuracy	Sensitivity	Specificity
**0.5**	88.9	90.6	86.2	87.1	86.7	87.5
**0.6**	89.0	91.3	85.3	87.7	87.9	87.5
**0.7**	88.8	91.7	84.1	87.7	88.8	86.7
**0.8**	88.5	92.3	82.5	**88.1**	**90.0**	**86.2**
**0.9**	88.5	93.4	80.4	86.9	91.2	82.5
